# Microglial Characterization in Transient Human Neurodevelopmental Structures

**DOI:** 10.1159/000528911

**Published:** 2023-01-05

**Authors:** David A. Menassa, Janja Kopić, Alisa Junaković, Ivica Kostović, Željka Krsnik

**Affiliations:** ^a^The Queen's College, University of Oxford, Oxford, UK; ^b^Croatian Institute for Brain Research, School of Medicine, University of Zagreb, Zagreb, Croatia

**Keywords:** Microglia, Human neurodevelopment, Subpial granular layer, Subplate zone, Connectivity

## Abstract

Human neurodevelopment is characterized by the appearance, development, and disappearance or transformation of various transient structures that underlie the establishment of connectivity within and between future cortical and subcortical areas. Examples of transient structures in the forebrain (among many others) include the subpial granular layer and the subplate zone. We have previously characterized the precise spatiotemporal dynamics of microglia in the human telencephalon. Here, we describe the diversity of microglial morphologies in the subpial granular layer and the subplate zone. Where possible, we couple the predominant morphological phenotype with functional characterizations to infer tentative roles for microglia in a changing neurodevelopmental landscape. We interpret these findings within the context of relevant morphogenetic and neurogenetic events in humans. Due to the unique genetic, molecular, and anatomical features of the human brain and because many human neurological and psychiatric diseases have their origins during development, these structures deserve special attention.

## Introduction

Human brain development is characterized by the expansion, reorganization, disappearance, or transformation of transient structures crucial to morphogenesis and function. This transience is necessary for the development of connectivity pathways and future brain regions [1]. For example, the subplate zone resolves by late gestation in humans, although part of SP neurons remains incorporated in the adult white matter as interstitial neurons [2]. In the ganglionic eminence and the subventricular and ventricular zones which are highly proliferative during gestation, transience ceases when proliferation ceases, and the remnants are incorporated into striatum and the neocortical wall, respectively. Mechanisms underlying disappearance or transformation of a structure may include cell death, pruning, or the retraction of exuberant axons [3].

In the forebrain, the subpial granular layer (SPGL) [5] and the subplate zone are transient structures that are well-developed in humans compared to other primates [4, 5, 6]. The subplate is an extracellular matrix (ECM)-rich compartment that receives afferents crucial for the establishment of thalamocortical and intracortical connectivities [2]. The subplate is also an area receiving migrating neurons from the intermediate zone to populate the cortical plate. The SPGL is thought to contribute stem cells with an interneuronal fate destined to reach the marginal zone (MZ) and support the excitation/inhibition balance of the future layer I [5, 7]. Many human neurological and psychiatric diseases have their origins during development. The human brain has unique genetic, molecular, and anatomical features [4]; therefore, these transient structures require special focus.

The neurodevelopmental landscape is shaped by morphogenetic, neurogenetic, and histogenetic events. Neocortical neurogenesis is subject to morphogenetic and neurogenetic gradients that support the projection neuron identity acquisition [8, 9]. Radial glia are pivotal in these processes as they form neuronal precursors but also the scaffold that facilitates neuronal migration. Radial glia also yield astrocytes [10]. Oligodendrocytes shape connectivity pathways through myelination from around 26 postconceptional weeks (pcw) until adolescence [11, 12]. Therefore, both of these neuroectodermal-derived cell types participate in neocortical expansion and the establishment of connectivity.

Microglia are mesoderm-derived glial cells that begin colonizing the brain rudiment from the 4th pcw at the very onset of many morphogenetic and neurogenetic events [12, 13]. The extent to which these cells participate in developmental events is known from rodents: microglia are required for guiding interneurons to their correct position in the cortex and the refinement of dopaminergic axons [14]; microglia restrict the progenitor pool by the phagocytosis of neuronal precursors [15] and prune supernumerary synapses [16]. In human development, ascribed microglial roles include the clearance of apoptotic cells in the telencephalon [17], the formation of white matter tracts such as the corpus callosum [18], and the tracking of migrating neurons in the frontal lobe [19]. Microglial spatiotemporal dynamics undulate within critical windows during early fetal, midfetal, and early postnatal life in the telencephalon [13]. These undulations are associated with the appearance of transient structures such as the cortical plate and the subplate. Though much remains to be done to investigate how microglial dynamics interact with the transient circuitry, it is clear that microglia are present in transient structures and participate in developmental processes [12].

Microglial morphology varies by the transient layer during brain development. We have previously documented the variability in morphologies observed in the telencephalic wall [13]. Microglia tend to be amoeboid when they arrive to the brain rudiment, develop a leading process to migrate tangentially or radially (to the pial surface) to reach their final destination, develop a proliferative core to expand the population size, or karyolyse to die in order to refine the population [13]. Phagocytic microglia develop pouches in their processes or at a late stage of phagocytosis are sizeable and amoeboid [20]. Microglia perform immune surveillance by retracting and protracting their processes while their cell body remains stationary [21]. Microglia are ramified or bipolar when in the steady-state and have reached their destined layer or clustered particularly around developing white matter tracts [12, 13]. This is not an exhaustive list of observed morphologies. However, microglial phenotypic pleiotropy is likely linked to a specific function be it the expansion of the population, the refinement of the neurodevelopmental landscape by axonal or synaptic pruning, the clearing of debris, the refinement of the population, or neuroinflammation [22, 23].

Here, we describe morphological microglial states in two transient structures that are well-developed in humans: the SPGL [22, 23] and the subplate zone [22, 23]. The SPGL is present in primates, and rodents do not have clearly recognizable SPGL [22, 23]. We interpret these morphological differences in light of existing neurodevelopmental events. We draw on these findings to suggest putative microglial roles in particular processes relevant to connectivity development. We hope that this morphological description of microglia in these transient structures serves as a basis for future more molecularly oriented studies.

## Materials and Methods

### Human Tissues

We selected 20 postmortem tissues aged between 12 and 33 pcw for this study (online suppl. Table 1; for all online suppl. material, see www.karger.com/doi/10.1159/000528911). Ethical approval was granted by the School of Medicine, University of Zagreb to use the Zagreb Brain Collection. The study was conducted according to the guidelines of the Declaration of Helsinki and approved by the Ethics Committee of School of Medicine University of Zagreb (protocol number 380-59-10106-15-168/314). Part of the human fetal material was provided by the Joint MRC/Wellcome Trust grant #099175/Z/12/Z Human Developmental Biology Resource. Exclusion criteria were congenital abnormalities, genetic disorders, brain trauma, periventricular leukomalacia, and hypoxic ischemic encephalopathy in the perinatal cases. Additional exclusion criteria were infection, brain trauma, psychiatric or neurological disease.

### Transient Structures of Interest

Anatomical delineation was performed using Nissl and periodic acid-Schiff reagent Alcian blue reactions to identify boundaries and ECM-rich compartments as previously described [2, 5, 7]. Our focus was the dorsal frontal telencephalic wall.

**Fig. 1:**
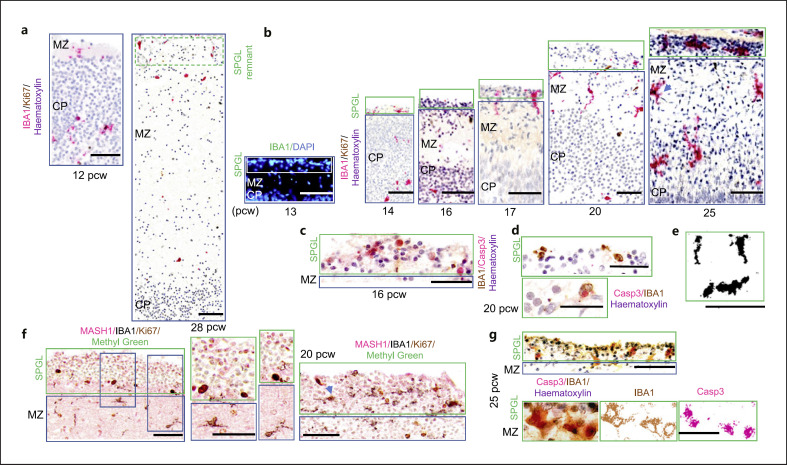
Mapping of microglia in the SPGL across human gestation. Overall, microglia primarily adopt transient morphologies in the SPGL. **a** Limits of when we can see the SPGL - at 12 pcw (left, scale bar, 200 μm), we do not see it, and at 28 pcw very little of it is left (right, scale bar, 500 μm). It appears first at 13 pcw as shown in (**b**) first column. **b** 13–25 pcw mapping of microglia in the SPGL; scale bar, 150 μm. **c** At 16 pcw, cell death can be observed with very few microglia visible; scale bar, 75 μm. **d** Microglial cells in the SPGL (top) at 20 pcw with an example of a microglial cell in proximity to a dying cell (bottom); scale bar, 50 μm. **e** Variability in microglial morphologies in the SPGL: horizontal tangential or vertical toward the MZ; scale bar, 25 μm. **f** Triple labeling of microglia (IBA1^+^, black), interneuronal progenitors (MASH1^+^, magenta), and proliferation (Ki67^+^, brown) at 20 pcw in the SPGL. The arrow points to a proliferating microglial cell (IBA1^+^Ki67^+^); scale bar, 100 μm. **g** Microglial death in the lower portion of the SPGL (IBA1^+^Casp3^+^); scale bar, 75 μm. CP, cortical plate; MZ, marginal zone; pcw, postconceptional weeks; SPGL, subpial granular layer.

The SPGL appears by the 13th pcw, is prominent toward mid-gestation, and resolves by the 28th pcw (Fig. 1a, b and [5, 7]). Similarly, the expanded subplate appears by the 12.5th pcw, reaches its prominence toward late gestation, and resolves by the 34th pcw [2].

### Golgi Labeling

Human brain hemispheres (12 and 14 pcw) were immersed in chrome-osmium fixative according to the Golgi rapid procedure as previously described [24]. Processing of tissues was according to the Stensaas (1967) modification of the Golgi-Del Rio Hortega labeling method [25]. After impregnation, the specimens were dehydrated, embedded in celloidin, and sectioned at 120–180 μm thickness as previously mentioned [26] until ready for imaging.

### Immunohistochemistry

Paraffin-embedded blocks were cut into thin sections of 8–10 μm on a microtome for immunohistochemistry and immunofluorescence. Brightfield immunohistochemistry and immunofluorescence were performed using antibodies against microglia with the following dilutions: rabbit (019–19741, Wako) or mouse (ab283319, Abcam) or goat (ab5076, Abcam) IBA1 at 1:200 and rabbit TMEM119 (ab185333, Abcam) at 1:1,000; neuronal stem cells with mouse MASH1 (14-5794-82, ThermoFisher) at 1:200. The first step was deparaffinization of formalin-fixed paraffin-embedded sections in xylol solution and rehydration in descending concentrations of diluted ethanol (100%, 96%, 70%). Antigen retrieval was done by heat-induced epitope opening using citric acid buffer (pH = 6.2). Thereafter, sections were pre-treated with methanol and hydrogen peroxide to block endogenous peroxidase and phosphatase activity. For triple labeling, two antibodies were done successively, and when the labeling was complete, the sections were requenched to block peroxidase activity and re-incubated with the third antibody. Sections were blocked with a solution of 5% BSA + Triton X-100 in 1X PBS and then incubated with primary antibodies overnight. The next day, secondary antibodies were applied using the ImmPRESS Duet kit (MP7724, Vector labs, UK) with anti-mouse epitopes visualized with DAB chromogen in brown and anti-rabbit epitopes visualized with alkaline phosphatase in magenta. For triple labeling, primary epitope detection was done using a biotinylated antibody (1:200) at pulled using streptavidin using the ABC kit before chromogenic development with a DAB and Nickel Kit (SK4100, Vector labs). Sections were counterstained with hematoxylin or methyl green and coverslipped with permanent mounting medium before imaging.

For immunofluorescence, we followed a similar workflow as our brightfield pipeline up until secondary antibody incubation whereby the secondary antibody was conjugated to a fluorophore at a dilution of 1:500 (Alexa Fluor 488 from Invitrogen, Germany). To block autofluorescence signals, we used TrueBlack lipofuscin quencher (Biotium, US). Mounting medium VECTASHIELD with DAPI was used to coverslip (Vector Laboratories).

### Brightfield and Immunofluorescence Slide-Scanning and Confocal Microscopy

Imaging was done using a high-resolution histological slide scanner Hamamatsu NanoZoomer 2.0 RS with a ×40 objective (numerical aperture of the lens = 0.75) at 0.45 μm × 0.45 μm pixel resolution. Fluorescent images were taken using the Hamamatsu LX2000 Lightning exciter (Hamamatsu Photonics, Japan). When the confocal was used, we used an Olympus FV3000 confocal microscope (numerical aperture = 1.35; 0.2 μm × 0.2 μm pixel resolution with the thickness of the imaged slice in one plane being 5 μm). We did not perform Z-stacks. For Golgi imaging, we also used the histological slide scanner Hamamatsu NanoZoomer 2.0 RS but with a ×20 objective (numerical aperture of the lens = 0.8) at 0.8 μm × 0.8 μm pixel resolution.

### Morphological Descriptive Assessment

We adopted the morphological classification based on our previously published documentation of encountered microglial morphologies during human development (supplementary in [13], summary in Introduction). In brief, microglial morphologies described were amoeboid, proliferative, dying, migrating (radial or tangential), ramified or bipolar, phagocytic or clustered. Thresholded images were obtained using Fiji image analysis software.

## Results

### Microglia Adopt Predominantly Transient Morphologies in the SPGL

The SPGL is not visible at 12 pcw, and by 28 pcw, very little of it remains (Fig. 1a). In the portion of the SPGL that we had in our samples, we describe here microglial colonization of that layer between 13 and 25 pcw. Consistent with previous reports, the SPGL appears first at 13 pcw (Fig. 1b) [5, 7]. Between 13 and 20 pcw, SPGL microglial morphology is consistent with either a migratory phenotype with a leading process pointing toward the MZ (Fig. 1b, e) or are horizontally aligned cells (Fig. 1b, e). No TMEM119^+^ cells are observed in the SPGL, and as we had previously reported, TMEM119 is most predominantly expressed at low levels in microglia during mid-gestation and positive cells are spatially restricted to the MZ and the ventricular zone but can be seen very sporadically in the middle layers [27]. Notably, non-microglial cell death is observed from 16 pcw (Fig. 1c). Microglial morphology consistent with a phagocytic phenotype positioned around dying cells (Fig. 1d) can also be seen but that tends to be at the border with the MZ. At 20 pcw and when the SPGL is at its most prominent, we see significant non-microglial proliferation with a high density of horizontally aligned IBA1^+^ cells in the vicinity and at the lower portion of the SPGL (Fig. 1f). Multiple interneuronal progenitors (MASH1^+^) (Fig. 1f) are seen at the upper portion of the SPGL. Between 13 and 19 pcw, proliferative microglial cells are usually located in the MZ (Fig. 1b) and rarely appear in the SPGL (Fig. 1g). From 20 pcw onward, we see few proliferative microglia in the SPGL (Fig. 1f). By 25 pcw, significant microglial death occurs in the SPGL (Fig. 1g), and the SPGL evanesces shortly thereafter. Ramified microglia are only observed in the MZ but never in the SPGL, and from 20 pcw, most microglia have a ramified morphology in the MZ (Fig. 1b, f). Overall, microglia adopt various morphologies in the SPGL, but the predominant morphology is transient and migratory.

### Microglial Morphological Diversity Follows the Stages of Subplate Development

**Fig. 2:**
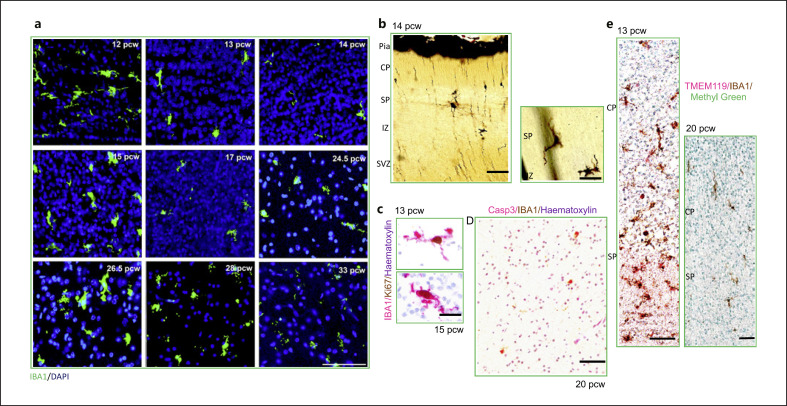
Mapping of microglial morphologies in the human subplate across human gestation. Overall, microglial morphological diversity follows the stages of subplate development. **a** Diversity of microglial morphologies in the subplate from its appearance at 12 pcw until the time of its resolution at 32–33 pcw. Microglia are largest at 12 pcw and as the subplate expands and evanesces, can be migratory, amoeboid, or ramified; scale bar, 100 μm. **b** Representative Golgi images showing microglial cells radially orientated in the subplate and tangentially orientated in the intermediate zone; scale bar, 50 μm. **c** Representative images of microglial proliferation; scale bar, 25 μm. **d** Cell death (IBA1^+^Casp3^+^) at 20 pcw of microglia in the human subplate; scale bar, 100 μm. **e** Transient expression of TMEM119 at the earliest appearance of the subplate at 13 pcw, and then, this is lost until late gestation. We also show here a representative 20 pcw absence of TMEM119 expression in the subplate; scale bar, 75 μm. CP, cortical plate; IZ, intermediate zone; MZ, marginal zone; pcw, postconceptional weeks; SVZ, subventricular zone; VZ, ventricular zone.

Microglial morphological diversity is according to the stages of subplate development between 12 and 33 pcw (Fig. 2a). Microglia are horizontally aligned and are largest at the very early stages of subplate formation (12–13 pcw) (Fig. 2a). The subplate is the location of intensive microglial proliferation which we had previously reported [27] (Fig. 2c). As the subplate expands, we start seeing microglial morphologies consistent with a migratory phenotype (Fig. 2a). As the subplate expands further, more morphologies can be distinguished including ramified cells particularly at the border between the subplate and the cortical plate (Fig. 2a, b), radially migrating (Fig. 2a), and more ramified cells tiling the space toward 33 pcw (Fig. 2a). Striking microglial death is detected in the subplate from 20 pcw (Fig. 2d) which is consistent with the trough following the expansion wave in the subplate that we had previously documented [27]. Microglial parenchymal marker TMEM119 was only detected transiently in the subplate at 13 pcw (Fig. 2e). The signal was lost for the remainder of gestation in this layer except for its consistent expression in the MZ, the earliest maturing layer, that we had previously reported [27]. Overall, microglial morphologies in the subplate compartment are very diverse, and we interpret these in the subsequent section.

## Discussion and Conclusion

We describe here the diversity of microglial morphologies encountered in transient brain structures that are well-developed in humans: the SPGL and the subplate zone. The SPGL originates from distinct sites of the basal forebrain with the earliest stages being paleocortical in origin and the later stages descending from the subventricular zone of the ganglionic eminences [7]. The heterogeneity of the SPGL cell populations has been documented, and in contrast to others, we are able to add to the repertoire of cells existing in that transient layer, microglia, which we detected throughout. From a neurogenetic context, the SPGL is speculated to provide signals for cortical folding [7]. The SPGL is a key compartment for tangentially migrating small neurons into the MZ, and microglial morphology is aligned with the orientation of these cells (Fig. 1b). The SPGL also generates some Cajal-Retzius cells, perhaps accounted for by some of the proliferation we observe at 20 pcw (Fig. 1f). It is not surprising to see dying cells (Fig. 1d) or amoeboid microglia near dying cells (Fig. 1d) as some Cajal-Retzius cells will start dying from mid-gestation onward. Some microglial cells will exit the SPGL and populate the MZ (Fig. 1b, e), while others are programmed to die as the layer evanesces (Fig. 1g) and speculatively as part of the refinement phase for the microglial population in the MZ similarly to what we had previously reported [27]. All these events including a contingent of ingrowing fibers and neurons failing to reach their destination make the SPGL an ideal morphogenetic ground for microglial cells.

The subplate compartment is the thickest and most voluminous transient layer in the fetal cortical wall and is composed of postmigratory and migratory neurons, growing axonal plexuses and synaptic and non-synaptic intercellular contacts in an ECM [2]. The origin of the subplate is debated, but it is agreed that subplate neurons are the first to form in the human neocortex [2, 28, 29]. Microglial morphologies appear to follow the stages of subplate development. Microglial cell morphology is aligned with neurogenetic and histogenetic processes within the subplate compartment. These include receiving afferents necessary for establishing cortical and subcortical connectivities, early synaptogenesis, and the subplate being on the migratory route for waves of neurons of different origins attempting to get to the cortical plate [2, 28].

Because we have described microglial morphological diversity in these transient zones, there are limitations to this approach, and further work is required to elucidate the signals between developing neurons and microglia to clarify the nature of the interaction. This small-scale study is useful to test hypotheses for further molecularly orientated studies.

In conclusion, microglial cells in the SPGL and the subplate zone show morphological pleiotropy which likely depends on the signals they receive from each morphogenetic, histogenetic, and neurogenetic process that helps in shaping the developing cortex. Microglia follow the development of these compartments and are likely involved in the sublaminar organization and reorganization of these evanescing structures. Future work is needed to identify the relevance of microglia in transient circuits to the origin of neurodevelopmental disorders.

## Statement of Ethics

Ethical approval was granted from the Ethics Committee of the School of Medicine, University of Zagreb (protocol number 380-59-10106-15-168/314). The study was conducted according to the guidelines of the Declaration of Helsinki. Part of the human fetal material was provided by the Joint MRC/Wellcome Trust grant #099175/Z/12/Z Human Developmental Biology Resource.

## Conflict of Interest Statement

All the authors declare no conflict of interest.

## Funding Sources

This work was supported by the “Research Cooperability” Program of the Croatian Science Foundation funded by the European Union from the European Social Fund under the Operational Programme Efficient Human Resources 2014–2020 PSZ-2019-02-4710 (ZK). Research was co-financed by the Scientific Centre of Excellence for Basic, Clinical, and Translational Neuroscience (project “Experimental and clinical research of hypoxic-ischemic damage in perinatal and adult brain”; GA KK01.1.1.01.0007 funded by the European Union through the European Regional Development Fund). This work was also supported by the Croatian Science Foundation project DOK-2020-01-5029 (A.J.).

## Author Contributions

D.A.M., Z.K., and I.K. conceived the study. D.A.M., J.K., and A.J. conducted the experiments, scanned the slides, and produced the photomicrograhps. All the authors contributed to the writing of the manuscript.

## Data Availability Statement

Data are available upon request from the lead and corresponding authors. All data generated or analyzed for this study are included in this article and its online supplementary material. Further inquiries can be directed to the corresponding authors. Data are generated from large human brain development scans (GBs in size), and access to these can be arranged by contacting the authors.

## Supplementary Material

Supplementary data
