# Supplementary material for: Microglial Characterization in Transient Human Neurodevelopmental Structures
Source: Dev Neurosci. 2023 Jan 5;45(1):1–7. doi: 10.1159/000528911 (PMC10015752; doi:10.1159/000528911)
Supplement: Supplementary file 1 — Supplementary data [file dne-0045-0001-s01.docx]

**Supplementary Table 1:** Case demographics

| **Case** | **GA** | **PCW** | **Sex** |  |  | | | **Cause of Death** | | **Histology** |
| --- | --- | --- | --- | --- | --- | --- | --- | --- | --- | --- |
| Specimen 1 | 14 | 12 | n/k |  | |  |  | Medical abortion | | Normal |
| Specimen 2 | 14 | 12 | F |  | |  |  | Medical abortion | | Normal |
| Specimen 3 | 15 | 13 | n/k |  |  | | | Medical abortion | | Normal |
| Specimen 4 | 16 | 14 | n/k |  |  | | | Medical abortion | | Normal |
| Specimen 5 | 16 | 14 | M |  |  | | | Medical abortion | | Normal |
| Specimen 6 | 17 | 15 | F |  |  | | | Medical abortion | | Normal |
| Specimen 7 | 18 | 16 | M |  |  | | | Medical abortion | | Normal |
| Specimen 8 | 18 | 16 | M |  |  | | | Medical abortion | | Normal |
| Specimen 9 | 19 | 17 | M |  |  | | | Spontaneous abortion | | Normal |
| Specimen 10 | 22 | 20 | M |  |  | | | Spontaneous abortion | | Normal |
| Specimen 11 | 23 | 21 | F |  |  | | | Spontaneous abortion | | Normal |
| Specimen 12 | 25 | 23 | M |  |  | | | Spontaneous abortion | | Normal |
| Specimen 13 | 25 | 23 | M |  |  | | | Stillbirth | | Normal |
| Specimen 14 | 26 | 24.5 | F |  |  | | | Septicemia (early neonatal sepsis) | | Normal |
| Specimen 15 | 27 | 25 | F |  |  | | | Stillbirth | Normal | |
| Specimen 16 | 27 | 25 | F |  |  | | | Placental abruption | Normal | |
| Specimen 17 | 28 | 26.5 | F |  |  | | | Placental abruption | Normal | |
| Specimen 18 | 28 | 26 | M |  |  | | | n/k | Normal | |
| Specimen 19 | 30 | 28 | F |  |  | | | Perinatal asphyxia | Normal | |
| Specimen 20 | 35 | 33 | n/k |  |  | | | Placental abruption | Normal | |
| Total number of cases shown is n = 20; *F:* female; *GA:* gestational age; *M:* male; *n/k:* not known; *PCW*: postconceptional week. | | | | | | | | | | |
